# Tumor-Derived Exosomal RNA From Fine-Needle Aspiration Supernatant as a Novel Liquid Biopsy for Molecular Diagnosis of Cancer

**DOI:** 10.3389/pore.2022.1610344

**Published:** 2022-08-05

**Authors:** Guorong Li, Dongdong Liu, Pascale Flandrin, Yang Zhang, Claude Lambert, Nora Mallouk, Michèle Cottier

**Affiliations:** ^1^ Department of Digestive Surgery and Urology, North Hospital, CHU Saint-Etienne, Saint-Etienne, France; ^2^ Department of Laboratory Science, The Second Affiliated Hospital of Guangzhou University of Chinese Medicine, Guangzhou, China; ^3^ Laboratory of Molecular Biology, North Hospital, CHU Saint-Etienne, Saint-Etienne, France; ^4^ Guangzhou HopeTech Biological Technology Co., Ltd., Guangzhou, China; ^5^ Section of Flow Cytometry, Immunology Laboratory, North Hospital, CHU Saint-Etienne, Saint-Etienne, France; ^6^ Center of Electronic Microscopy, CMES, Faculty of Medicine, University Jean Monnet, Saint-Etienne, France; ^7^ Laboratory of Cytopathology, North Hospital, CHU Saint-Etienne, Saint-Etienne, France

**Keywords:** exosomes, pancreatic adenocarcinoma, liquid biopsy, FNA supernatant, molecular diagnosis, glypican 1

## Abstract

**Background:** We hypothesized that the fine needle aspiration (FNA) supernatant from tumor might contain tumor-derived exosomes. The objective of this pilot study was to test if tumor-derived exosomal RNA could be found in FNA supernatants for molecular diagnosis of cancer.

**Methods:** 10 FNA samples from pancreatic tumor were included. After the routine recuperation of cellular material by centrifugation, the cell-free Cytolyt liquid was collected instead of being discarded. 10 ml Cytolyt was used to isolate the exosomes. Transmission electronic microscopy (TEM) was used to examine the presence of exosomes. The exosomal marker CD63 was analyzed by flow cytometry. The exosomal RNA was extracted. RT-qPCR was performed to detect the GAPDH and the tumor marker of glypican 1 gene expression.

**Results:** TEM confirmed the presence of exosomes from FNA supernatants. Flow cytometry showed a strong positive expression of exosome marker CD63. The concentration of exosomal RNA ranged from 18.81 to 354.75 ng/μl with an average of 81.76 ng/μl. The average exosomal RNA quantity was 1390.01 ng (range from 319.77 to 6030.75 ng) with an average 260/280 ratio of 2.12. GAPDH was detectable in all samples. Exosomal glypican 1 was detected in all samples of pancreatic ductal adenorcarcinomas (3/3) and absent from benign cystic samples (3/3). Furthermore, exosomal glypican 1 was positive in one sample with a non-contributive cytology and in one sample in which no malignant cell was found.

**Conclusion:** This is the first report that the supernatants from FNA biopsy may contain tumor-derived exosomal RNA. These tumor-derived exosomes from FNA may provide a new liquid biopsy for the molecular diagnosis of cancer.

## Introduction

Pancreatic ductal adenocarcinoma (PDAC) is one of major leading cause of cancer-related deaths in the United States and in Europe. Most of patients have an unresectable tumor at the time of diagnosis due to locoregional invasion or distant metastasis [1–3]. The diagnosis by tissue biopsy is needed in the clinical circumstances. The diagnosis of a benign tumor can lead to initiate a conservative treatment or an imaging follow-up. A preoperative tissue diagnosis is performed by using image-guided fine needle aspiration (FNA) or needle biopsies. Percutaneous pancreatic FNA cytologic sampling has been described since 1975 [4]. The sensitivity of percutaneous imaging-guided FNA biopsy in the diagnosis of pancreatic masse ranges from 62% to 100% [5]. Nowadays, EUS-guided FNA (EUS-FNA) has been performed frequently to make a preoperative diagnosis and to stage pancreatic carcinoma. Although some studies suggest an improved sensitivity, experts agree that interpreting pancreatic EUS-FNA samples can be inherently difficult. The interpretation is challenging especially when cellular materials are scant and/or bloody, as often observed on EUS-FNA samples. Non-diagnostic samples (inadequate or equivocal diagnoses) from pancreas and other organs for EUS-FNA samples range from 11% to 30%. A molecular marker that can help in differentiating the malignant cells of pancreatic adenocarcinoma from the benign cells of reactive ductal epithelium would be very utile [6].

Liquid biopsy is a recent topic [7]. Liquid biopsy is based on the detection of tumor markers in body fluid, mostly in the blood, urine, saliva, or another fluid. Liquid biopsy is considered to be preferable to tissue biopsy because it is less invasive. Exosomes are membrane-bound vesicles secreted by all types of cells into the extracellular space. It has been found that exosomes play an important role in physiological and pathological processes. Many studies have shown that exosomes participate in cancer development and cancer progression. Exosomal research has become a hot subjet in the research area of biomarkers because exosomes carry specific proteins, lipids and nucleic acids of their cellular origins [8]. More importantly, exosomes provide a convenient source of potential disease biomarkers because they are ample in accessible body fluids. In fact, exosome has rapidly become as a major source of liquid biopsy.

In clinical routine, FNA supernatants are discarded. FNA supernatant contains the liquid from tumor, making it an attractive source of liquid biopsy. Tumor DNA has been found in FNA supernatant which can be used for mutation analysis in solid pancreatic tumor [9–11]. In this pilot study, we hypothesize that FNA supernatant may contain tumor-derived exosomes. We established a technique to isolate tumor-derived exosomes from FNA supernatant and tested if tumor-derived exosomes from FNA could be used as a molecular tool to diagnose cancer.

## Materials and Methods

### Patients

The pancreatic FNA samples were randomly included. The indication for a pancreatic FNA biopsy was that patient had either a cystic or a solid pancreatic mass. Four patients had a solid tumor, two patients had a mixed cystic and solid tumor and four patients had a cystic tumor. The tumor size ranged from 2.0 to 6.1 cm. There were three males and seven females. Their age ranged from 49 to 84 years old. An experienced clinician performed the EUS-FNA of pancreatic lesion as a clinical routine. As a routine practice, the cellular materials of FNA biopsy were put in 30 Cytolyt solution, which was sent to cytology laboratory. As a laboratory routine, the Cytolyt solution was centrifuged at 1500 rmp for 5 min to obtain the cells. The cellular material was used for cytological diagnosis. The supernatant was collected instead of being discarded. The supernatant was stocked in −20°C until the isolation of exosome. The standard technique of ThinPrep with the Papapicolau coloration was carried out by laboratory technicians. The cytological diagnosis was performed as a laboratory routine examination. The cellularity was routinely classed as satisfactory cellular material, scant cellular material and non-cellular material according to our previous publication [12]. The collection of cytoponction samples for analysis was performed in the framework of cytology biobank which was reviewed and approved by the local IRB (protocol code: DC20121659).

### Isolation of Exosomes From Fine Needle Aspiration Supernatant

10 ml FNA supernatant was utilized to isolate the exosomes by using a commercial exosome isolation solution (Hope Tech Biotechnology Co. Ltd., Guangzhou). Briefly, 2 ml of isolation solution was put into the supernatant. The mixture of solution was vortexed and placed in a refrigerator overnight. The mixture was then centrifuged at 3000 g for 30 min at 4°C to collect the exosomes.

### Transmission Electronic Microscopy of Exosomes From Fine Needle Aspiration Supernatant

Transmission electron microscopy was used to examine the exosomes resuspended in 50 µl PBS. 10 µl of exosome solution was put on grids and left to adhere for 20 min. The grids were washed three times by using distilled water. The exosomes were negatively stained with uranyl acetate. Then exosomes were observed using a Hatachi electron microscopy.

### Flow Cytometry of Exosomal Marker CD63

FCM was utilized to check the exosomal marker CD63. We added 1 µl of Aldehyde/sulphate latex beads (A37304, ThermoFisher) to 200 µl of exosome suspension. The solution was well mixed. Then we added 800 µl of PBS into the solution, and the mixture was left to incubate for 2 h at room temperature on a rotary wheel. The beads were collected by centrifugation at 4000 g for 5 min, and the supernatant was discarded. The beads were washed by the addition of 1 ml PBS and then were centrifuged at 4000 g for 5 min. Finally, the beads were suspended in PBS. The antibody of anti-human CD63-FITC (IM1165U, Beckman Coulter) or the antibody of an isotype control (A07795, Beckman Coulter) was added according to the standard protocol for FCM. The antibody was incubated for 30 min at dark. After twice washing with PBS, the staining was analyzed by using a conventional FCM.

### Extraction of Exosomal RNA

We performed the total RNA extraction by using miRNeasy Micro Kit according to the manufacture instructions with some modifications (Qiagen S.A.). Briefly, 500 µl Qiazol solution (GITC-containing buffer) was added to dissolve the exosomes. Then 100 µl chloroform was added. The mixture was vortexed and centrifuged at 14,000 rmp at 4°C for 15 min. The supernatant was secured and 1.5 volume of 100% ethanol was added. The extraction column (Qiagen S.A.) was used to extract the total RNA from the mixture. The column was washed, respectively by RWT buffer and by REP buffer. Finally, the toal RNA was eluted in 17 µl RNase free water. The RNA was then quantified by using Nanodrop. The size distribution of exosomal RNA was analyzed by using a 2100 Bioanalyzer (Agilent Technologies). The RNA specimens were kept in –80°C until use.

### RT-qPCR of Tumor Markers in Exosomes

We performed one-step RT-qPCR by using Taqman Reverse Transcription Kit (Invitrogen). We performed PCR in 25 µl reaction mixture containing 3 μl RNA, 0.5 µl of primers/probe and 12.5 µl PCR mix. After a denaturing temperature at 95°C for 10 min, 40 cycles were carried out with denaturing temperature at 95°C for 15 s, annealing temperature at 60°C for 20 s and extension at 72°C for 34 s. A positive control of universal carcinoma RNA standard and a negative control (PCR mix without RNA) were performed in each round of PCR. To examine the quality of extracted RNA, we checked the GAPDH gene expression. The primers and probes for GAPDH and glypican 1 were the commercial products of Life Technologies (TaqMan^®^ Gene Expression Assay, Invitrogen-Life Technologies). The cycle threshold (Ct) value was set up by using the detection software SDS v2.0.1 (Applied Biosystem). GAPDH was utilized as the housekeeping gene. A Ct value less than 35 was considered as positive.

## Results

### Cytological Diagnosis

There were four solid tumors, two heterogene tumors and four cystic tumors. Table 1 shows the cellularity and cytological diagnosis. There were six samples with satisfactory cellularity, two samples with scant cellularity and two samples with non-cellular material. Pancreatic adenocarcinoma was found in three samples. Two samples were diagnosed with no cancer cells. Three samples were marked with a cyst. Finally, two samples were non-contributive.

**TABLE 1:** Cytological characteristics.

Patient no.	Sex	Age	Tumor type	Cellularity	Cytological Diagnosis
1	Female	72	Solid	Satisfactory	PDAC
2	Female	70	Solid	Satisfactory	PDAC
3	Female	51	Solid	Satisfactory	PDAC
4	Female	84	Solid	Non-cellular	Not contributive
5	Male	75	Heterogene	Satisfactory	No malignant cells
6	Male	64	Heterogene	Satisfactory	No malignant cells
7	Female	49	Cystic	Scant	Cyst
8	Female	71	Cystic	Satisfactory	Cyst
9	Female	69	Cystic	Scant	Cyst
10	male	57	Cystic	Non-cellular	Not contributive

### Transmission Electronic Microscopy and Flow Cytometry of Exosomes From Fine Needle Aspiration Supernatant

**FIGURE 1:**
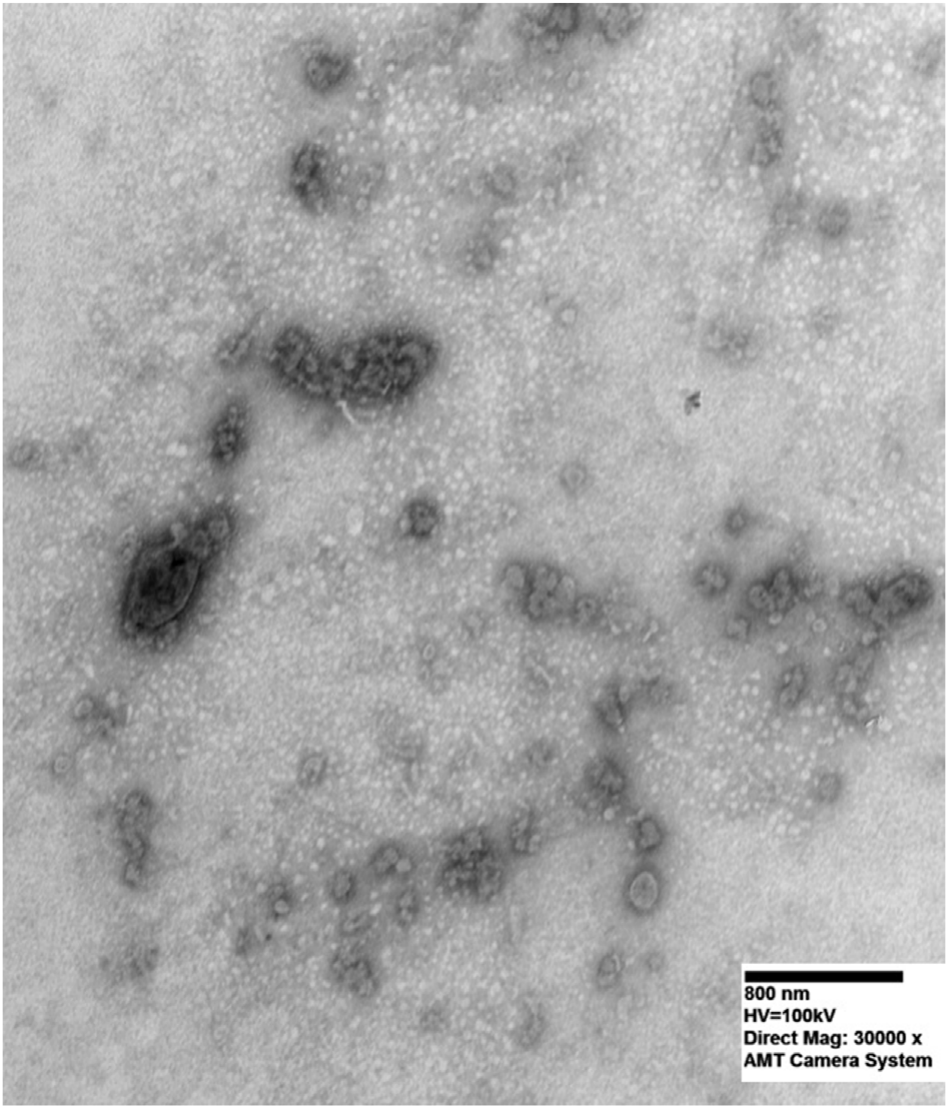
Transmission electron microscopy of exosomes in FNA supernatant. Typical exosome morphology of cup-shaped vesicles was observed along with aggregated vesicles. Image was taken under 30,000 magnification.

**FIGURE 2:**
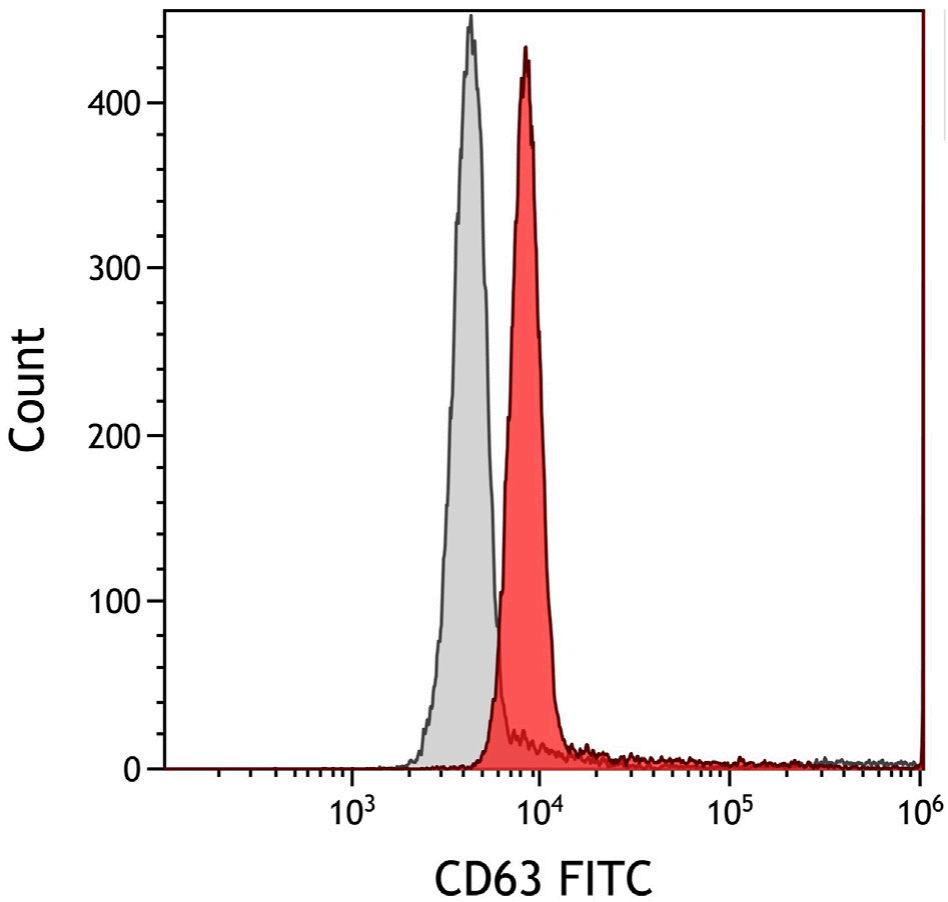
Flow cytometry analysis of exosome marker CD63. The exosomes showed a strong positive expression for exosome marker CD63.

Figure 1 shows the exosomes under electronic microscopy. TEM analyses indicated that the isolated exosomes from FNA supernatant were within the expected size range and with typical exosome morphology (Figure 1). The cup-shaped vesicles were observed together with aggregated vesicles. The abundant supernatant exosomes in cancer patients were observed. The size of exosomes was about 100–150 nm. Two experiments were performed with similar results. FCM demonstrated that the exosomes had a strong positive for exosomal marker CD63 (Figure 2).

### Exosomal RNA Quantity and Quality

**FIGURE 3:**
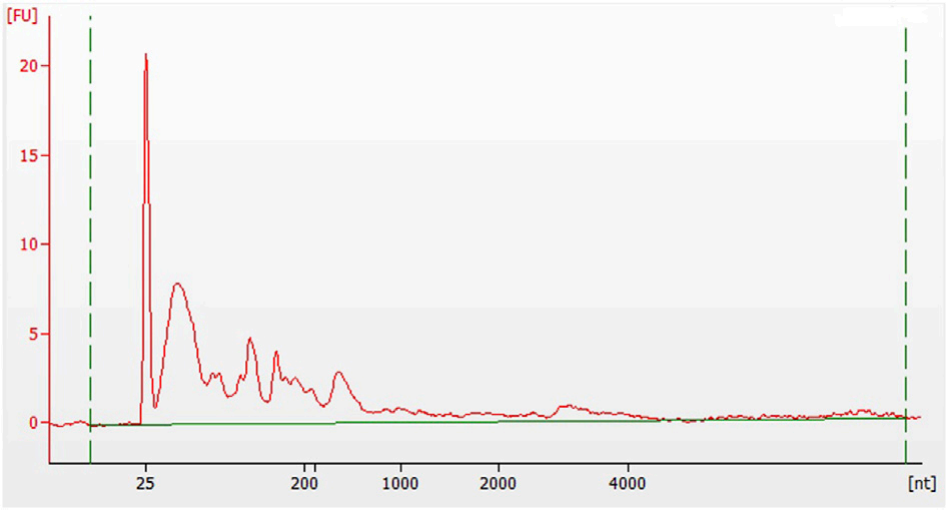
Fragment analysis of exosomal RNA. Electrophoretic analysis of a sample demonstrated that the value of DV200 (the percentage of RNA fragments >200 nucleotides) was less than 50%. FU stands for the fluorescence unit, and nt for the nucleotide.

Table 2 shows the RNA quantity and quality. The concentration of RNA ranged from 18.81 to 354.75 ng/μl with an average of 81.76 ng/μl. The RNA quantity ranged from 319.77 to 6030.75 ng with an average of 1390.01 ng. The 260/280 ratio ranged from 1.76 to 2.75 with an average of 2.12. The quantity and quality of RNA in supernatant exosomes were excellent. We found that RNA quantity in scant and non-cellular samples was abundant. An example of RNA size distribution is shown in Figure 3. The size distribution of the exosomal RNA varied greatly among the samples. The electrophoretic analysis showed that the value of DV200 (the percentage of RNA fragments >200 nucleotides) was less than 50% in the majority of samples.

**TABLE 2:** Exosomal RNA quantity and quality.

Patient no.	Concentration RNA (ng/µl)	Ratio 260/280	Quantity RNA (ng)	Glypican 1 expression
1	77.80	2.07	1322.60	Positive
2	85.91	2.04	1460.47	Positive
3	46.10	2.05	783.70	Positive
4	43.36	2.07	737.12	Positive
5	96.27	2.09	1636.59	Positive
6	22.44	2.75	381.48	Negative
7	41.17	1.80	699.89	Negative
8	18.81	2.05	319.77	Negative
9	31.04	1.76	527.68	Negative
10	354.75	1.92	6030.75	Negative

### Tumor Marker Glypican 1 Expression From Exosomes

All samples had a positive GAPDH expression. Table 2 shows the RT-qPCR result of glypican 1. Tumor marker glypican 1 was detected in all samples of pancreatic adenorcarcinomas and absent from benign samples. Furthermore, exosomal glypican 1 was positive in one sample of scant cellularity with a non-contributive cytology and in one sample in which no malignant cell was found.

## Discussion

Exosomes are recently utilized as a novel and important source of liquid biopsy. Exosomes carry the same RNA markers as the cells of their origin. Tumoral RNA markers may be enriched in tumor-derived exosomes. In this pilot study, we found that FNA supernatant contained abundant tumor-derived exosomal RNA with an excellent quantity. More importantly, we found that exosomal RNA quantity in scant and non-cellular samples was abundant.

Clinically, FNA is a pratical tool of obtaining tissue material. With the increasing use of molecular markers, the tissue material is not only used for routine diagnosis but also for molecular tests. It is recommended to utilize optimally the tissue material [13–16]. However, it may be difficult to fulfill this task since the tissue materials can be limited while the demand is incresaing. Several researchers have found that FNA supernatants contain tumor DNA, which can be used for molecular testing in pancreatic cancer [7–9]. The purpose is to find the tumor DNA mutations before the targeted therapy. Tumor DNA is also found in FNA supernatant of FNA samples from thyroid and lung tumors [17, 18]. Ye et al found that discarded supernatants from thyroid FNA needle rinses could provide large amounts of DNA for the detection of BRAF mutations even when the corresponding FNA smear was sparsely cellular [18]. However, no tumor-derived exosome from FNA biopsy has been studied so far for the diagnostic purpose. We decided to seek for the exosomes in FNA supernatant as a novel liquid biopsy for cancer diagnosis.

Cytological analyses are often confounded by sampling failure and interobserver variability. As other FNA samples, there are two major problems for pancreatic FNA biopsy. One problem is for the lack of tissue material and another is the difficulty in interpretation of morphology. The traditional tumor markers in the biopsy samples are used for the purpose of diagnosis. In this study, we confirmed the presence of exosomes in pancreatic FNA supernatant. Glypican 1 is a heparan sulphate proteoglycan in cell surface. It is overexpressed in many solid tumors. Its role is to regulate tumor development, progression and metastasis [19]. Many reports support that glypican 1 is a new marker for pancreatic adenorcarcinoma. Recently, Melo et al found that exosomes positive for glypican 1 could detect all stages of pancreatic adenocarcinomas with a high sensitivity and specificity [20]. Frampton et al found that glypican 1 was highly expressed on circulating exosomes in early and late stages of pancreatic cancers, when compared to normal controls and those with benign pancreatic diseases [21]. However, it may be difficult to detect the exosomal glypican 1 in blood for the purpose of diagnosis because of the composition heterogeneity of blood exosomes. In this pilot study, we found that exosomal glypican 1 could be detected in FNA supernatants. All pancreatic adenocarcinomas had positive exosomal glypican 1 while benign tumors were negative. Furthermore, we got some evidence that the exosome glypican 1 could help cytological diagnosis. In patient n°4, the cytology was non-contributive because of non-cellularity, but its exosome RNA was abundant and its exosme glypican 1 was positive. This result may suggest a pancreatic adenocarcinoma. In patient n°5, no cancer cell was found although its cellularity was satisfactory. But the exosomal glypican 1 was positive in this sample, which may suggest a pancreatic adenocarcinoma. We agree that it would be interesting to look for the final pathological diagnosis of these two patients if they were operated on. But our goal was to prove the existence of tumor-derived exosomal RNA in FNA supernatants. We believe that exosomal glypican 1 in pancreartic FNA supernatant will become a powerful molecular adjunct to cytological diagnosis of pancreatic adenocarcima.

The pancreatic FNA Cytolyt supernatants are routinely discarded. It is very easy to make a suggestion of using them for molecular tests. Our results indicate that FNA supernatants provide tumor-derived exosomes that can be utilized directly for nucleic acid extraction. Theoretically, RNA is protected from the degradation by exosome membrane. To our knowledge, it is the first report that a FNA supernatant contains abundantly the exosomal RNA. A FNA biopsy is not only useful for a tissue diagnosis, but it is also an excellent tool for a liquid biopsy. It means an immediate translation of exosome technique into clinical use of cytological diagnosis. However, this is a pilot study. The major limitation is that the number in this study was limited. Large studies are needed to confirm our results and its diagnostic value. Another limitation is that only one tumor marker of glypican 1 was tested. We think that other molecular tests are also possible by using the exosomes from FNA supernatant, i.e., DNA-based molecular tests.

## Conclusion

This is the first report that the supernatants from FNA biopsy may contain tumor-derived exosomes. These tumor-derived exosomes may provide a new source of liquid biopsy for the molecular diagnosis of cancer.

## Data Availability

The original contributions presented in the study are included in the article/supplementary material, further inquiries can be directed to the corresponding authors.
